# Systematic Review of Serum Biomarkers in Traumatic Brain Injury

**DOI:** 10.7759/cureus.17056

**Published:** 2021-08-10

**Authors:** Khashayar Mozaffari, Dillon Dejam, Courtney Duong, Kevin Ding, Alexis French, Edwin Ng, Komal Preet, Alyssa Franks, Isabelle Kwan, H. Westley Phillips, Dennis Y Kim, Isaac Yang

**Affiliations:** 1 Neurosurgery, Ronald Reagan University of California Los Angeles Medical Center, Los Angeles, USA; 2 Neurosurgery, David Geffen School of Medicine, University of California, Los Angeles, USA; 3 Neurosurgery, University of California, Los Angeles, USA; 4 Biomedical Sciences, Harbor University of California Los Angeles Medical Center, Los Angeles, USA

**Keywords:** traumatic brain injury, biomarkers, glial fibrillary acidic protein, s100 calcium binding protein b, neuron specific enolase

## Abstract

Traumatic brain injury (TBI) is responsible for the majority of trauma-related deaths and is a leading cause of disability. It is characterized by an inflammatory process involved in the progression of secondary brain injury. TBI is measured by the Glasgow Coma Scale (GCS) with scores ranging from 15-3, demonstrating mild to severe brain injury. Apart from this clinical assessment of TBI, compendiums of literature have been published on TBI-related serum markers.Herein we create a comprehensive appraisal of the most prominent serum biomarkers used in the assessment and care of TBI.The PubMed, Scopus, Cochrane, and Web of Science databases were queried with the terms “biomarker” and “traumatic brain injury” as search terms with only full-text, English articles within the past 10 years selected. Non-human studies were excluded, and only adult patients fell within the purview of this analysis. A total of 528 articles were analyzed in the initial search with 289 selected for screening. A further 152 were excluded for primary screening. Of the remaining 137, 54 were included in the final analysis. Serum biomarkers were listed into the following broad categories for ease of discussion: immune markers and markers of inflammation, hormones as biomarkers, coagulation and vasculature, genetic polymorphisms, antioxidants and oxidative stress, apoptosis and degradation pathways, and protein markers. Glial fibrillary acidic protein(GFAP), S100, and neurons specific enolase (NSE) were the most prominent and frequently cited markers. Amongst these three, no single serum biomarker demonstrated neither superior sensitivity nor specificity compared to the other two, therefore noninvasive panels should incorporate these three serum biomarkers to retain sensitivity and maximize specificity for TBI.

## Introduction and background

Traumatic brain injury (TBI) is responsible for the majority of trauma-related deaths and is a leading cause of disability [1]. It is characterized by an inflammatory process involved in the progression of secondary brain injury [2]. Clinically, it is measured by the Glasgow Coma Scale (GCS), a scoring system that relies on categories of eye-opening, verbal response, and motor response [3]. Scores ranging from 15-13 suggest mild injury, 12-9 imply moderate injury, and 8-3 indicate severe injury. Apart from the clinical assessment of TBI, a compendium of literature has been published on TBI-related serum markers [1,3-5]. Studies have reported elevated levels of inflammatory cytokines such as IL-6 and IL-8 in severe TBI [4,6]. In addition, TBI can cause dysregulation of the endocrine system, thus leading to an array of symptoms [2,7,8]. Lastly, protein markers such as S100 calcium binding protein B (S100B) have been extensively studied [9-13]. The proteins, enzymes, degradation products, amino acids, chemokines, hormones, and free nucleotides vary in terms of efficacy and utility depending on the degree of brain injury. 

Although TBI has been an active topic of research, the practical application of serum biomarkers in this pathology is yet to be elucidated. The purpose of this article is to evaluate the most current human biomarkers found in the serum that are used in the hospital setting, and to determine which ones hold the most utility for determining the presence and severity of brain injury.

## Review

Materials and methods

This study adhered to the Preferred Reporting Items for Systematic Reviews and Meta-Analyses (PRISMA) guidelines. PubMed, Scopus, Cochrane, and Web of Science databases were queried using “biomarker” and “traumatic brain injury” as search terms. Only articles and data reported in English were included. Abstract-only texts, book chapters, animal studies, articles in languages other than English, and irrelevant articles were excluded.

Results

**Figure 1:**
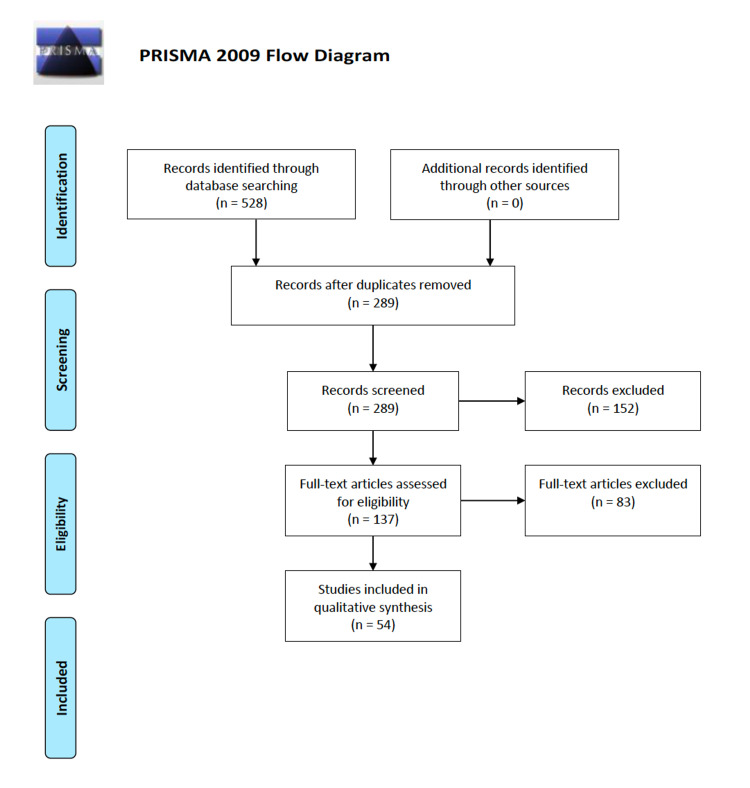
PRISMA flowchart showing the exclusion and selection process of the 54 articles included in the qualitative review PRISMA: Preferred Reporting Items for Systematic Reviews and Meta-Analyses

In total, 528 articles were analyzed in the initial search. 289 articles were screened after duplicate removal, and 152 articles were excluded for meeting one or more of the exclusion criteria (animal studies, book chapters, etc.). Subsequently, 137 articles were assessed for full-text eligibility, of which 83 were excluded for either not directly addressing TBI serum biomarkers, or if they dealt exclusively with spinal injury or general trauma. Lastly, 54 studies were selected for qualitative analysis (Figure 1). Serum biomarkers were listed into the following broad categories for ease of discussion: immune markers and markers of inflammation, hormones as biomarkers, coagulation and vasculature, genetic polymorphisms, antioxidants and oxidative stress, apoptosis and degradation pathways, and protein markers. 

Discussion

Immune Markers and Markers of Inflammation

In terms of the immune response, numerous potential biomarkers were encountered across the innate and adaptive arms. One study demonstrated that serum and cerebrospinal fluid (CSF) IL-6 levels were significantly elevated in fatal TBI patients compared to non-fatal TBI patients [4]. Another study reported high serum IL-8 levels to be associated with moderate and severe cerebral hypoperfusion in severe TBI [6]. Serum mannose binding lectins (MBL) are key components of innate immunity. Compared to their healthy cohorts, patients with severe TBI had notable elevations in serum MBL. Multivariate analysis showed this to be closely related to GCS scores with a receiver operating curve confirming its predictive value [1]. Galectin-3, a member of the lectin family and involved in microglial activation, was demonstrated to have elevated plasma concentrations in TBI patients, and was also an indicator for hospital mortality [14]. In contrast, lower levels of Ficolin 3, an activator of the lectin complement pathway, is associated with poorer outcomes in TBI patients [5].

Regarding the inflammatory process, a few markers were discovered upon literature search. Plasma levels of adiponectin, a marker of inflammation, were found to be elevated in patients with TBI compared to controls, and were shown to be independent predictors of mortality and unfavorable outcomes [15]. High-mobility group box 1 (HMGB1) is a cytokine and marker of inflammation that was shown by Wang et al. to be an independent predictor for one-year mortality in patients with TBI [16]. HMGB1 translocates from the nucleus to the cytoplasm in early TBI, then enters phagocytic microglia in later time points. Aside from serving as a biomarker, this may be a promising therapeutic target [17].

Hormones as Biomarkers

Damage to regions of the brain can lead to disruptions in regulation of the endocrine system and axes across numerous organ systems. TBI can be evaluated by looking for common hormones. For example, increased plasma copeptin levels were associated with mortality after TBI [7].

Other hormones of interest include nesfatin-1 and resistin. Nesfatin-1 is related to inflammation and is an independent predictor for hospital mortality. Its concentrations in plasma are related to the severity of TBI, and it has the potential to become a reliable prognostic marker for these injuries [2]. Resistin, commonly referred to as adipocyte-secreted factor (ADSF), is an adipose tissue-specific secretory factor. After TBI, plasma resistin levels rise starting at six hours then peak at 24 hours. It was found through logistic regression and receiver operating curve analysis that resistin is an independent predictor for one-month mortality of patients [8].

Coagulation and Vasculature

Plasma-Thombospondin-1 (TSP-1), an anti-angiogenic factor [18], has been shown to increase in head trauma and is associated with one-week and six-month mortality outcomes of severe traumatic brain injury. It was also predictive for the development of pituitary dysfunction in cases of mild TBI [19]. Demonstrating favorable specificity and sensitivity, this marker is promising for detecting late pituitary dysfunction in patients with TBI. Patients with brain injury had increased plasma Vascular Adhesion Protein 1 (sVAP-1) in accordance with the severity of TBI. A cut off of 8.61 nmol/mL/hr was set, with patients reaching levels higher than this showing a 25% increased mortality rate [20].

Protein Markers

S100B is an intracellular calcium binding protein found in astrocytes and is one of the most widely studied biomarkers for TBI [9-13,21-23]. Serum S100B concentration in the acute phase of TBI has been shown to negatively correlate with resting state brain connectivity, as determined by fMRI [24]. One study has demonstrated that the addition of S100B testing into management guidelines for TBI may prove to be cost-effective and lower CT usage rates [25]. S100B serum concentrations have been shown to change significantly over timescales that are relevant for early determination of prognosis [26]. Interestingly, patients undergoing surgery for spine or lower extremity fractures experienced a significant increase in serum S100B concentration compared to preoperative concentrations [27]. External ventricular drain placement has also been shown to affect S100B levels, although this time in the CSF, and serum S100B levels greater than 0.7 μg/dL have been observed to correlate with 100% mortality for TBI and subarachnoid hemorrhage [28]. S100B concentration has been shown to not display clinically significant seasonal variation [29].

One study demonstrated that S100B levels below 0.105 μg/L were predictive of normal cranial CT findings after minor head injury in patients above 65 years old as well as patients being treated with platelet aggregation inhibitors, and the same study demonstrated that the use of S100B testing can reduce cranial CT scan and hospital readmission rates by up to 30% [30]. Combining S100B testing with radiologic studies has been shown to improve the prediction of clinical outcomes [31]. S100B levels have been shown to vary depending on the type and number of lesions in TBI [32]. Secondary increases in S100B, as low as less than 0.05 μg/L, are correlated with the development of clinically significant secondary radiologic findings [33]. Calcagnile and colleagues demonstrated that S100B testing can be used in the context of mild TBI patients with alcohol intoxication, however the same study also showed that the use of S100B testing in older patients may be limited by poor specificity leading to only a small decrease in CT scanning [34]. Lange and colleagues demonstrated that S100B testing was more accurate in sober patients compared to patients intoxicated with alcohol [35]. 24-hour serum S100B levels may serve as a screening tool for the early detection of patients at risk for brain death after severe TBI [36]. S100B may be effective as a treatment monitoring tool for TBI, as one study showed that S100B levels decreased after hyperosmolar therapy [37].

It was suggested that S100B samples obtained within 12 hours of the traumatic injury are of less prognostic value compared to S100B samples obtained 12-36 hours after the injury [38]. Urine S100B levels have also been suggested to provide prognostic value comparative to serum S100B levels [39]. Combining S100B levels with glial fibrillary acidic protein (GFAP) levels has been shown to result in accurate prediction of one-year mortality after TBI [40]. One study suggested that allelic variation in the APOE gene may play a role in the interpretation of S100B levels [31]. Evaluation of S100A12 variants of S100B levels may be helpful in prognostic prediction for severe TBI patients [19]. S100A12 mRNA expression is significantly increased in peripheral blood of TBI patients within 24 hours of injury compared to controls [41]. Similar to the calcium modulating effects of S100B, neurogranin (NGRN) is a neuronal protein that regulates calmodulin availability and has potential as a tumor marker. Serum NGRN concentrations were found to be significantly higher in acute TBI patients compared to controls [42].

Tau protein, also known as MAPT, is a protein that plays a role in neuronal development, axonal stabilization, and neuronal polarity. It was noted that both serum and CSF levels of MAPT may be considered a biomarker for TBI after postmortem examination revealed elevated MAPT levels even when macroscopic examination revealed no visible trauma, implying that some damage may have still occurred [43]. Rubenstein et al. demonstrated that hypo-phosphorylated tau protein (P-tau) plasma levels and P-tau/total-tau ratios outperformed total-tau as diagnostic and prognostic markers of TBI, and compared with total-tau levels alone, P-tau levels and P-tau/total-tau ratios show more robust and sustained elevations among patients with chronic TBI [44]. Serum cleaved tau protein levels were also shown to be significantly higher in severe TBI compared to controls [45]. Total tau levels correlated well with clinical and radiologic variables of TBI [46]. Poor outcomes in severe TBI were associated with higher serum tau protein levels [47].

Neuron specific enolase (NSE) containing microparticles were found to be higher in TBI compared to healthy controls [48], and its levels were found to be significantly higher for patients who died or had poorer outcomes [49]. Treatment of moderate TBI patients with memantine resulted in a significant reduction of serum NSE levels and improvement in GCS scores [50]. Thelin et al. suggested that NSE may not be as accurate or as clinically useful compared to S100B [51]. Another report countered that high NSE levels at 72 hours demonstrated high prediction powers of mortality [31]. NSE has also been demonstrated to be higher in patients with TBI and secondary hypoxia compared to normoxic TBI patients [52]. The initial rise and peak of NSE levels were higher in non-survivors than survivors of TBI [40]. Allelic variation of the APOE gene may also play a role in the elevation of NSE in severe TBI patients [53]. One study found that, compared to S100B, elevations in NSE were more closely associated with prediction of brain death after severe TBI [21]. Neurofilament Heavy Subunit (NF-H) is a nervous system-specific protein that is released from tissue after TBI, and serum phosphorylated NF-H at 24 hours after injury was determined to be a good predictive marker of death at six months after injury [54].

## Conclusions

Of the serum biomarkers mentioned within this review, GFAP, S100, and NSE, were the most prominent and frequently cited. Numerous studies sought to compare the efficacy of one over the other in prospective and retrospective fashions with mixed results. Lacking a clear mandate, noninvasive panels should incorporate these three serum biomarkers to retain sensitivity and maximize specificity for TBI. In the future, further research is needed to develop the other biomarkers discussed in the groupings above, with an emphasis on developing an area under the receiver operating curve to provide standardized comparison across markers.

## References

[1] Yu W, Le HW, Lu YG, Hu JA, Yu JB, Wang M, Shen W (2015). High levels of serum mannose-binding lectins are associated with the severity and clinical outcomes of severe traumatic brain injury. Clin Chim Acta.

[2] Wu GQ, Chou XM, Ji WJ (2016). The prognostic value of plasma nesfatin-1 concentrations in patients with traumatic brain injury. Clin Chim Acta.

[3] Teasdale G, Jennett B (1976). Assessment and prognosis of coma after head injury. Acta Neurochir (Wien).

[4] Ondruschka B, Schuch S, Pohlers D, Franke H, Dreßler J (2018). Acute phase response after fatal traumatic brain injury. Int J Legal Med.

[5] Pan JW, Gao XW, Jiang H, Li YF, Xiao F, Zhan RY (2015). Low serum ficolin-3 levels are associated with severity and poor outcome in traumatic brain injury. J Neuroinflammation.

[6] Stein DM, Lindell AL, Murdock KR (2012). Use of serum biomarkers to predict cerebral hypoxia after severe traumatic brain injury. J Neurotrauma.

[7] Dong XQ, Huang M, Yang SB, Yu WH, Zhang ZY (2011). Copeptin is associated with mortality in patients with traumatic brain injury. J Trauma.

[8] Dong XQ, Yang SB, Zhu FL, Lv QW, Zhang GH, Huang HB (2010). Resistin is associated with mortality in patients with traumatic brain injury. Crit Care.

[9] Ondruschka B, Pohlers D, Sommer G, Schober K, Teupser D, Franke H, Dressler J (2013). S100B and NSE as useful postmortem biochemical markers of traumatic brain injury in autopsy cases. J Neurotrauma.

[10] Wolf H, Frantal S, Pajenda GS, Salameh O, Widhalm H, Hajdu S, Sarahrudi K (2013). Predictive value of neuromarkers supported by a set of clinical criteria in patients with mild traumatic brain injury: S100B protein and neuron-specific enolase on trial: clinical article. J Neurosurg.

[11] DeFazio MV, Rammo RA, Robles JR, Bramlett HM, Dietrich WD, Bullock MR (2014). The potential utility of blood-derived biochemical markers as indicators of early clinical trends following severe traumatic brain injury. World Neurosurg.

[12] Goyal A, Failla MD, Niyonkuru C, Amin K, Fabio A, Berger RP, Wagner AK (2013). S100b as a prognostic biomarker in outcome prediction for patients with severe traumatic brain injury. J Neurotrauma.

[13] Topolovec-Vranic J, Pollmann-Mudryj MA, Ouchterlony D (2011). The value of serum biomarkers in prediction models of outcome after mild traumatic brain injury. J Trauma.

[14] Shen YF, Yu WH, Dong XQ (2016). The change of plasma galectin-3 concentrations after traumatic brain injury. Clin Chim Acta.

[15] Shen LJ, Yang SB, Lv QW (2014). High plasma adiponectin levels in patients with severe traumatic brain injury. Clin Chim Acta.

[16] Wang KY, Yu GF, Zhang ZY, Huang Q, Dong XQ (2012). Plasma high-mobility group box 1 levels and prediction of outcome in patients with traumatic brain injury. Clin Chim Acta.

[17] Gao TL, Yuan XT, Yang D (2012). Expression of HMGB1 and RAGE in rat and human brains after traumatic brain injury. J Trauma Acute Care Surg.

[18] Wang KK, Yang Z, Yue JK (2016). Plasma anti-glial fibrillary acidic protein autoantibody levels during the acute and chronic phases of traumatic brain Injury: a transforming research and clinical knowledge in traumatic brain injury pilot study. J Neurotrauma.

[19] Feng MJ, Ning WB, Wang W (2018). Serum S100A12 as a prognostic biomarker of severe traumatic brain injury. Clin Chim Acta.

[20] Lin Z, Han M, Li H, Luo H, Zhang Y, Luo W (2011). Soluble vascular adhesion protein-1: decreased activity in the plasma of trauma victims and predictive marker for severity of traumatic brain injury. Clin Chim Acta.

[21] Böhmer AE, Oses JP, Schmidt AP (2011). Neuron-specific enolase, S100B, and glial fibrillary acidic protein levels as outcome predictors in patients with severe traumatic brain injury. Neurosurgery.

[22] Vos PE, Jacobs B, Andriessen TM (2010). GFAP and S100B are biomarkers of traumatic brain injury: an observational cohort study. Neurology.

[23] Blyth BJ, Farhavar A, Gee C (2009). Validation of serum markers for blood-brain barrier disruption in traumatic brain injury. J Neurotrauma.

[24] Thompson WH, Thelin EP, Lilja A, Bellander BM, Fransson P (2016). Functional resting-state fMRI connectivity correlates with serum levels of the S100B protein in the acute phase of traumatic brain injury. Neuroimage Clin.

[25] Calcagnile O, Anell A, Undén J (2016). The addition of S100B to guidelines for management of mild head injury is potentially cost saving. BMC Neurol.

[26] Ercole A, Thelin EP, Holst A, Bellander BM, Nelson DW (2016). Kinetic modelling of serum S100b after traumatic brain injury. BMC Neurol.

[27] Wolf H, Krall C, Pajenda G, Hajdu S, Widhalm H, Leitgeb J, Sarahrudi K (2016). Preliminary findings on biomarker levels from extracerebral sources in patients undergoing trauma surgery: potential implications for TBI outcome studies. Brain Inj.

[28] Kellermann I, Kleindienst A, Hore N, Buchfelder M, Brandner S (2016). Early CSF and serum S100B concentrations for outcome prediction in traumatic brain injury and subarachnoid hemorrhage. Clin Neurol Neurosurg.

[29] Henriksson AE (2016). S100B and the influence of seasonal variation. Scand J Clin Lab Invest.

[30] Thaler HW, Schmidsfeld J, Pusch M (2015). Evaluation of S100B in the diagnosis of suspected intracranial hemorrhage after minor head injury in patients who are receiving platelet aggregation inhibitors and in patients 65 years of age and older. J Neurosurg.

[31] Olivecrona Z, Bobinski L, Koskinen LO (2015). Association of ICP, CPP, CT findings and S-100B and NSE in severe traumatic head injury. Prognostic value of the biomarkers. Brain Inj.

[32] Wolf H, Frantal S, Pajenda G, Leitgeb J, Sarahrudi K, Hajdu S (2015). Analysis of S100 calcium binding protein B serum levels in different types of traumatic intracranial lesions. J Neurotrauma.

[33] Thelin EP, Nelson DW, Bellander BM (2014). Secondary peaks of S100B in serum relate to subsequent radiological pathology in traumatic brain injury. Neurocrit Care.

[34] Calcagnile O, Holmén A, Chew M, Undén J (2013). S100B levels are affected by older age but not by alcohol intoxication following mild traumatic brain injury. Scand J Trauma Resusc Emerg Med.

[35] Lange RT, Iverson GL, Brubacher JR (2012). Clinical utility of the protein S100B to evaluate traumatic brain injury in the presence of acute alcohol intoxication. J Head Trauma Rehabil.

[36] Egea-Guerrero JJ, Murillo-Cabezas F, Gordillo-Escobar E (2013). S100B protein may detect brain death development after severe traumatic brain injury. J Neurotrauma.

[37] Hendoui N, Beigmohammadi MT, Mahmoodpoor A (2013). Reliability of calcium-binding protein S100B measurement toward optimization of hyperosmolal therapy in traumatic brain injury. Eur Rev Med Pharmacol Sci.

[38] Thelin EP, Johannesson L, Nelson D, Bellander BM (2013). S100B is an important outcome predictor in traumatic brain injury. J Neurotrauma.

[39] Rodríguez-Rodríguez A, Egea-Guerrero JJ, León-Justel A (2012). Role of S100B protein in urine and serum as an early predictor of mortality after severe traumatic brain injury in adults. Clin Chim Acta.

[40] Gradisek P, Osredkar J, Korsic M, Kremzar B (2012). Multiple indicators model of long-term mortality in traumatic brain injury. Brain Inj.

[41] Petrone AB, Gionis V, Giersch R, Barr TL (2017). Immune biomarkers for the diagnosis of mild traumatic brain injury. NeuroRehabilitation.

[42] Yang T, Song J, Bu X (2016). Elevated serum miR-93, miR-191, and miR-499 are noninvasive biomarkers for the presence and progression of traumatic brain injury. J Neurochem.

[43] Olczak M, Niderla-Bielińska J, Kwiatkowska M, Samojłowicz D, Tarka S, Wierzba-Bobrowicz T (2017). Tau protein (MAPT) as a possible biochemical marker of traumatic brain injury in postmortem examination. Forensic Sci Int.

[44] Rubenstein R, Chang B, Yue JK (2017). Comparing plasma phospho tau, total tau, and phospho tau-total tau ratio as acute and chronic traumatic brain injury biomarkers. JAMA Neurol.

[45] Pandey S, Singh K, Sharma V (2017). A prospective pilot study on serum cleaved tau protein as a neurological marker in severe traumatic brain injury. Br J Neurosurg.

[46] Bogoslovsky T, Wilson D, Chen Y (2017). Increases of Plasma Levels of Glial Fibrillary Acidic Protein, Tau, and Amyloid β up to 90 Days after Traumatic Brain Injury. J Neurotrauma.

[47] Liliang PC, Liang CL, Weng HC, Lu K, Wang KW, Chen HJ, Chuang JH (2010). Tau proteins in serum predict outcome after severe traumatic brain injury. J Surg Res.

[48] Nekludov M, Bellander BM, Gryth D, Wallen H, Mobarrez F (2017). Brain-derived microparticles in patients with severe isolated TBI. Brain Inj.

[49] Žurek J, Fedora M (2012). The usefulness of S100B, NSE, GFAP, NF-H, secretagogin and Hsp70 as a predictive biomarker of outcome in children with traumatic brain injury. Acta Neurochir (Wien).

[50] Mokhtari M, Nayeb-Aghaei H, Kouchek M (2018). Effect of memantine on serum levels of neuron-specific enolase and on the Glasgow Coma Scale in patients with moderate traumatic brain injury. J Clin Pharmacol.

[51] Thelin EP, Jeppsson E, Frostell A, Svensson M, Mondello S, Bellander BM, Nelson DW (2016). Utility of neuron-specific enolase in traumatic brain injury; relations to S100B levels, outcome, and extracranial injury severity. Crit Care.

[52] Yan EB, Satgunaseelan L, Paul E (2014). Post-traumatic hypoxia is associated with prolonged cerebral cytokine production, higher serum biomarker levels, and poor outcome in patients with severe traumatic brain injury. J Neurotrauma.

[53] Olivecrona Z, Koskinen LO (2012). The release of S-100B and NSE in severe traumatic head injury is associated with APOE ε4. Acta Neurochir (Wien).

[54] Shibahashi K, Doi T, Tanaka S (2016). The serum phosphorylated neurofilament heavy subunit as a predictive marker for outcome in adult patients after traumatic brain injury. J Neurotrauma.

